# Changes in Circulating Extracellular Vesicles in Patients with ST-Elevation Myocardial Infarction and Potential Effects of Remote Ischemic Conditioning—A Randomized Controlled Trial

**DOI:** 10.3390/biomedicines8070218

**Published:** 2020-07-16

**Authors:** Paul M. Haller, Bernhard Jäger, Edita Piackova, Larissa Sztulman, Claudia Wegberger, Johann Wojta, Mariann Gyöngyösi, Attila Kiss, Bruno K. Podesser, Andreas Spittler, Kurt Huber

**Affiliations:** 1 3rd Department of Medicine, Cardiology and Intensive Care Medicine, Wilhelminenhospital, 1160 Vienna, Austria; bernhard.jaeger@meduniwien.ac.at (B.J.); dr.edita.piackova@gmail.com (E.P.); larissa.sztulman@gmail.com (L.S.); cwegberger@gmail.com (C.W.); kurt.huber@meduniwien.ac.at (K.H.); 2 Ludwig Boltzmann Institute for Cardiovascular Research, 1160 Vienna, Austria; johann.wojta@meduniwien.ac.at (J.W.); attila.kiss@meduniwien.ac.at (A.K.); bruno.podesser@meduniwien.ac.at (B.K.P.); 3 Faculty of Medicine, Sigmund Freud University, 1020 Vienna, Austria; 4 Core Facility, Medical University of Vienna, 1090 Vienna, Austria; andreas.spittler@meduniwien.ac.at; 5 Department of Internal Medicine II, Division of Cardiology, Medical University of Vienna, 1090 Vienna, Austria; mariann.gyongyosi@meduniwien.ac.at; 6 Department of Biomedical Research, Medical University of Vienna, 1090 Vienna, Austria

**Keywords:** extracellular vesicles, cardioprotection, remote ischemic conditioning, myocardial infarction, flow cytometry

## Abstract

(1) Background: Extracellular vesicles (EVs) have been recognized as a cellular communication tool with cardioprotective properties; however, it is unknown whether cardioprotection by remote ischemic conditioning (RIC) involves EVs. (2) Methods: We randomized patients with ST-elevation myocardial infarction (STEMI) undergoing primary percutaneous coronary intervention (PCI) to additionally receive a protocol of RIC or a sham-intervention. Blood was taken before and immediately, 24 h, four days and one month after PCI. Additionally, we investigated EVs from healthy volunteers undergoing RIC. EVs were characterized by a high-sensitive flow cytometer (Beckman Coulter Cytoflex S, Krefeld, Germany). (3) Results: We analyzed 32 patients (16 RIC, 16 control) and five healthy volunteers. We investigated platelet-, endothelial-, leukocyte-, monocyte- and granulocyte-derived EVs and their pro-thrombotic sub-populations expressing superficial phosphatidylserine (PS^+^). We did not observe a significant effect of RIC on the numbers of circulating EVs, although granulocyte-derived EVs were significantly higher in the RIC group. In line, RIC had not impact on EVs in healthy volunteers. Additionally, we observed changes of PS^+^/PEV, EEVs and PS^+^/CD15^+^ EVs irrespective of RIC with time following STEMI. 4) Conclusion: We provide further insights into the course of different circulating EVs during the acute and sub-acute phases of STEMI. With respect to the investigated EV populations, RIC seems to have no effect, with only minor differences found for granulocyte EVs.

## 1. Introduction

Changes in clinical practice during the last decades have led to a substantial decline in mortality of patients with ST-elevation myocardial infarction (STEMI) [1]. One major contributor comprises the timely restoration of blood flow in the occluded coronary artery, which is achieved most effectively by means of primary percutaneous coronary intervention (PCI) nowadays [2,3]. Of note, the resulting infarct size is strongly associated with morbidity and mortality following the acute event [4]. In this regard, the phenomenon of ischemia and reperfusion injury (IRI) describes an acute exacerbation of tissue damage upon reperfusion of the ischemic myocardium [5]. As reviewed previously, several interventions directly targeting IRI have failed upon being tested in large-scale clinical randomized-controlled trials so far [6]. Similarly, interventions (i.e., (stem) cell therapy) targeting at a reduction of the infarct-induced myocardial scare in order to raise cardiac function have failed so far as well [7,8]. Accordingly, there is still urgent need to address IRI and the development of heart failure following STEMI. 

Remote ischemic conditioning (RIC) describes a phenomenon when tissue is protected from IRI by performing so-called ischemic conditioning in a remote tissue or organ [9]. Although the exact mechanism of cardioprotection by RIC is still a matter of debate, studies conducted in different animal models of ischemia-reperfusion injury reported a profound cardioprotective effect [10]. Additionally, proof-of-principle trials in humans with acute myocardial infarction initially reported promising results as reviewed elsewhere [11]. In clinical practice, RIC may be conducted by application of a blood pressure cuff on a limb, strong inflation to introduce ischemia for several minutes, followed by deflation (reperfusion of the limb) and, commonly, three to five repetitions of this cycle. While this intervention provided a robust effect in several animal models as demonstrated previously [10], the potential effect in humans was questionable as shown by a meta-analysis of randomized-controlled trials [12]. In addition, a recently published adequately powered trial investigating the influence of RIC on cardiac death and hospitalization for heart failure came out neutral [13]. 

Recently, small lipid-bilayer-enclosed vesicles released by cells, and known as extracellular vesicles (EVs), have been shown to exert cardioprotection themselves [14,15,16]. EVs cargo proteins, lipids and micro ribonucleic acids between cells and are understood as a tool of cellular communication. This gives them a high potential as being biomarkers in several, including cardiovascular, diseases, as well being a potential therapeutic target [17,18,19,20,21]. Additionally, previous studies suggested an effect of RIC on circulating EVs, that was associated with cardioprotection [14,15,16]. These observations are based primarily on animal models, but data in humans are scarce. 

Therefore, we aimed to investigate the impact of RIC on circulating EVs during the acute and subacute phase in patients with STEMI in a pilot randomized, controlled trial.

## 2. Materials and Methods 

### 2.1. Patient Population

We performed a randomized-controlled trial including patients above the age of 18 years with first-ever STEMI who were scheduled to undergo immediate coronary angiography and primary PCI as appropriate. STEMI was defined in accordance with current guidelines [22]. In brief, patients with symptoms suggestive of angina pectoris or any equivalent (e.g., dyspnea) with concomitant ST-elevation on the ECG of at least 1 mm (2 mm in precordial leads) for at least 30 min, and no other differential diagnosis being more likely (e.g., aortic aneurysm), fulfilled inclusion criteria. We excluded patients with symptom onset prior to 8 h at the time of hospital presentation, known neurologic disorders (among others diabetic neuropathy), concomitant intake of drugs affecting the K_ATP_-channel (e.g., glibenclamide and nicorandil), patients with planned conservative management (i.e., not undergoing coronary angiography) and patients in cardiogenic shock or other situations making informed consent impossible. All patients gave written informed consent prior to enrolment. 

Additionally, we enrolled five healthy volunteers independently from the STEMI population. These participants were required to be free from any known comorbidity and were not allowed to use any medication on a regular basis. The study was performed according to the Declaration of Helsinki and Good Clinical Practice and was approved by the competent ethics committee of the City of Vienna (EK 16-009-0216 on 6 April 2016).

### 2.2. Study Interventions

STEMI patients scheduled for emergency coronary angiography were randomly assigned to undergo either additional RIC (group A; 4 cycles of upper left arm ischemia achieved by inflation of a blood pressure cuff up to 200 mHg for 5 min followed by 5 min deflation) or a sham-procedure (group B; application of a blood pressure cuff on the left upper arm without any inflation). Group allocation was derived from sequentially numbered and sealed envelopes, which were opened after written informed consent was obtained. The envelopes contained a computer-generated random group allocation. All study interventions started immediately after group assignment and were performed in parallel to routine clinical management, in order to not delay primary PCI. RIC or the sham procedure were immediately started after written informed consent and were also performed during PCI if necessary. Clinical and diagnostic assessment, as well as treatment was at the full discretion of the emergency care physician, intensive care and cardiology specialist and the PCI operator in charge, who all were not involved in the data collection and analysis of the trial.

Independently from the enrolment of STEMI patients, all participating healthy volunteers did undergo the same RIC protocol during a scheduled study visit. 

### 2.3. Study Parameters

Blood for analysis of EVs was taken in patients with STEMI at presentation directly after study inclusion (and before the start of RIC/sham treatment), within the first hour after PCI, as well as 24 h, 4 days and 1 month thereafter. For healthy volunteers, blood was taken immediately before and after conduction of the RIC protocol. Characterization and enumeration of EVs was performed as previously described [23,24] and is presented in detail in the online Supplementary Material. In brief, citrate-anticoagulated blood was centrifuged two times to obtain platelet free plasma (PFP), which was immediately frozen at −80° Celsius. For characterization, PFP was thawed in a 35° Celsius water bath for 10 min and labelled with anti-human monoclonal antibodies, followed by detection and characterization using a fluorescence-triggered detection protocol on a high-sensitive flow cytometer (Cytoflex S, Beckman Coulter, Krefeld, Germany). EVs are presented as number per microliter of PFP. 

**Figure 1:**
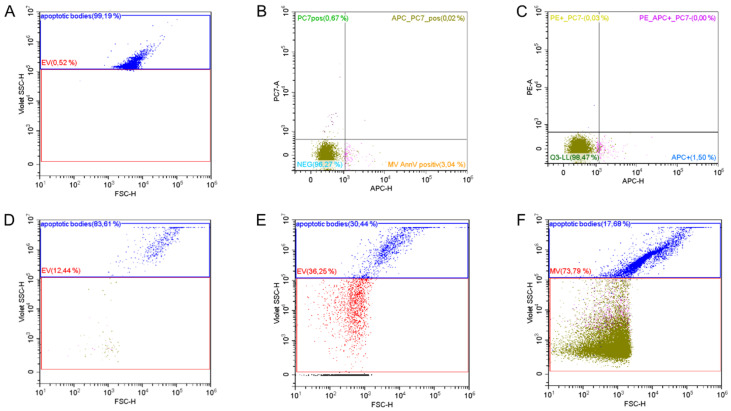
Methodological considerations for the detection of extracellular vesicles (EVs). (**A**) Setting the upper size limit using 1000 nm Silica beads in a violet-side-scatter (SSC-H)/forward scatter plot (FSC-H). Isotype controls for (Phycoerythrin Cyanin 7) PC7 (**B**) and (Phycoerythrin) PE (**C**) were used to set gates. (**D**) Washing steps with sterile water were performed before every measurement to assure a clean system and avoid spill-over. (**E**) Labelled EVs were destroyed using triton to confirm their presence (EV gate in absolute numbers: 155/µL plasma). In comparison, (**F**) provides a scatter-plot of a conventionally stained sample (EV gate absolute numbers: 1138 EVs/µL plasma). APC = Allophycocyanin

We used the following anti-human monoclonal antibodies in pre-defined combinations for EV characterization: CD41-PC7 (clone P2), CD54-PE (clone 84H10), CD146-PE (clone TEA 1/34), CD11b-PE (clone Bear1), all from Beckman Coulter (Krefeld, Germany), CD14-PE/Cy7 (clone HCD14), CD15-PE/Cy7 (clone W6D3) or CD31-PE (clone WM50) all from Biolegend (San Diego, CA, USA) for 90 min to achieve maximal antibody binding. Afterwards, samples were incubated with 0.083 µg bovine Lactadherin-Alexa 647 (Cell Systems, Troisdorf, Germany) and the intra-vesicular dye Calcein AM (Life Technologies, Carlsbad, CA, USA) for 30 min. Calcein AM is transported through the vesicle membrane into intact vesicles and becomes a strong green fluorophore after esterase-triggered conversion to calcein [23]. All incubation steps were performed at room temperature in the dark. The setup for EV characterization and control experiments are shown in Figure 1.

Additionally, we measured creatine kinase (CK) and cardiac troponin T at the above-mentioned times, as well as 2, 6, 12, 24 and 48 h after PCI. Peak CK values were used as a surrogate of final infarct size. All laboratory assessments, including EV analysis, were blinded to group allocation and laboratory and clinical data was merged after just after quality control of all parameters.

### 2.4. Statistics

Scale variables were tested for parametric distribution by investigating Q–Q plots and are presented as mean ± standard deviation, unless otherwise stated. Categorical variables are presented as absolute numbers and percent. Comparison of categorical variables between groups was performed using Chi2-test. Comparisons of scale variables over time were performed using linear mixed-models. In all models, patients were treated as random-effect to account for the repeated measurements design. Other variables were treated as fixed-effect. In a first step, models were built including an interaction term of time and group allocation to investigate potential differences between groups at different times. If the interaction term did not reach the level of significance or the plots did not suggest potential differences between groups, the interaction term was dropped from the model. Models were also compared using the Akaike information criterion and the model with the lowest value was chosen in case of uncertainties of model structure. We report fixed-effect estimates of the variables included in the model together with the 95% confidence intervals and the *p*-value. For all calculations we used “R 3.4.3” [25].

## 3. Results

From April 2016 to February 2018, we enrolled 37 patients, of which 32 patients (16 RIC, 16 controls) were included in the present analysis (Supplementary Figure S1). Baseline characteristics between groups were balance except for a history of hypertension, which was significantly more frequent in patients of the RIC group and accompanied by a significant higher proportion of ambulatory anti-hypertensive prescriptions at admission (Table 1). Peak cardiac troponin I and creatine kinase (CK) concentrations were not significantly different between groups (Table 1). 

### 3.1. Platelet-Derived EVs

**Figure 2:**
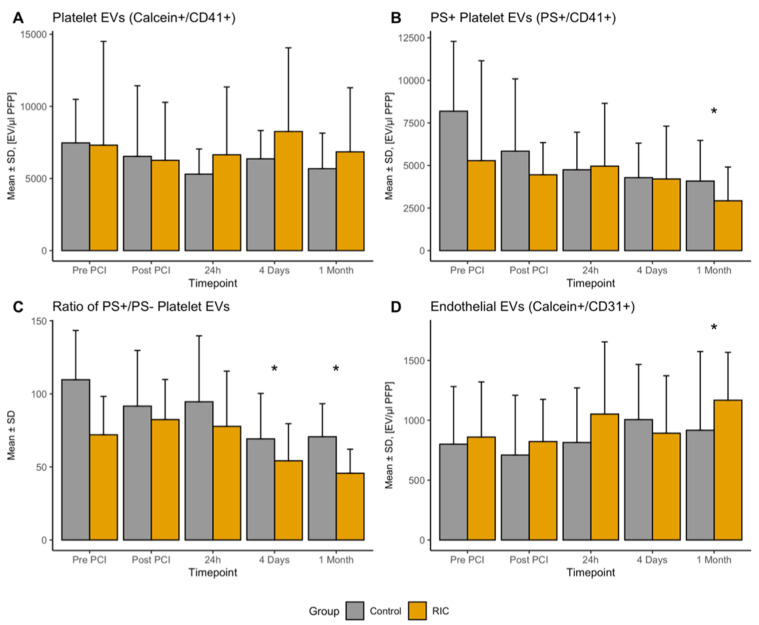
Number of circulating platelet-derived and endothelial EVs stratified by remote ischemic conditioning at all investigated times. (**A**) Provides results of all platelet-derived EVs, whereas (**B**) provided results for all pro-coagulatory platelet-derived EVs, which also express PS on their surface and their ratio is provided in (**C**). Endothelial EVs are provided in (**D**). All results are presented as mean ± standard deviation. * Marks statistically significant results at the specific time compared to “Pre PCI” derived from mixed-models. EV—extracellular vesicle; PCI—percutaneous coronary intervention; PS—phosphatidylserine; RIC—remote ischemic.

With respect to the influence of RIC on the number of circulating platelet-derived EVs (PEV, CalceinAM^+^/CD41^+^) during and after STEMI, we did not observe any difference (ln(PEV) −0.17 (95%CI −0.09–0.43), *p* = 0.18, Figure 2A). Additionally, there was no change over time (all time points *p* > 0.05). However, the baseline number of EVs was significantly associated with the further course of EVs across all investigated times (ln(PEV) 0.37 (95%CI 0.17–0.58), *p* < 0.001). With respect to pro-coagulatory PEVs, defined by the superficial expression of PS (PS^+^/CD41^+^), there was also no influence of RIC (ln(PS^+^/PEV) −0.09 (95%CI −0.4–0.26), *p* = 0.63, Figure 2B); however we observed a significant change over time with significantly less PS^+^/PEV after one month (ln(PS^+^/PEV) −0.3 (95%CI −0.6–0.07), *p* = 0.015). In line, the ratio of PS^+^ PEV and PS^−^ PEVs changed over time. As shown in Figure 2D, there were significantly less PS^+^ PEVs in relation to PS^−^ PEVs at four days (−25.7% (95%CI −38.3–−13.3), *p* < 0.001) and one month (−32.4% (95%CI −44.7–−20.1), *p* < 0.001) after PCI, respectively.

### 3.2. Endothelial-Derived EVs

Endothelial-derived EVs (EEVs) did not show a significant change during the acute phase after STEMI, but were significantly higher in the sub-acute phase one month after the acute event for the whole study population (270 (95% CI 21–521), *p* = 0.0359, Figure 2C). This was also evident if considering relative changes from baseline (81.6% (95% CI 27.2–136.0), *p* = 0.0036). RIC had no influence on the absolute numbers of EEVs (110 (95% CI −79–298), *p* = 0.2603). Additionally, the fraction of PS^+^ EEVs was not significantly altered at the investigated time points or by RIC (all *p* > 0.05).

### 3.3. Leukocyte-Derived EVs

**Figure 3:**
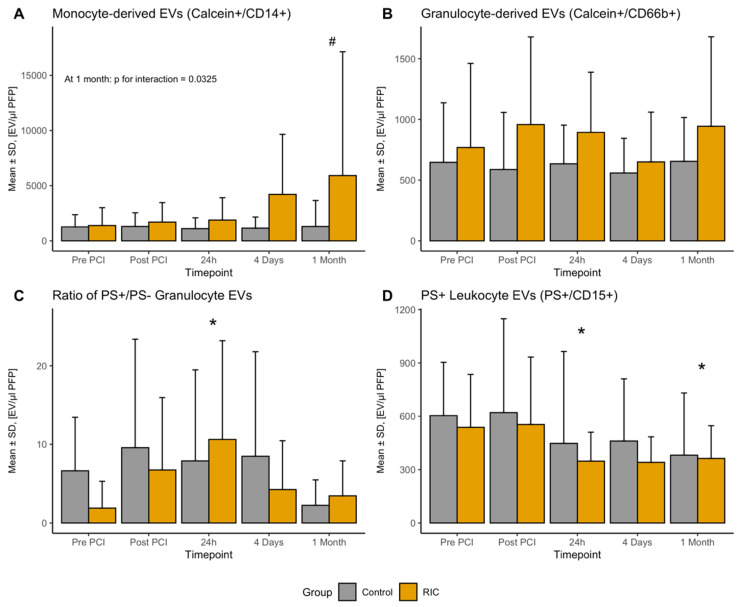
Number of circulating leukocyte-derived EVs stratified by remote ischemic conditioning at all investigated times. (**A**) Provides results of all monocyte-derived EVs, (**B**) shows the number granulocyte-derived EVs. The ratio of PS positive and negative granulocyte derived EVs is presented in (**C**). (**D**) Provides results of PS^+^ leukocyte (CD15^+^) EVs. All results are presented as mean ± standard deviation. * marks statistically significant results at the specific time compared to “Pre PCI” derived from mixed-models. # marks a statistically significant interaction term comparing RIC and control at the specific time. EV—extracellular vesicle; PCI—percutaneous coronary intervention; PS—phosphatidylserine; RIC—remote ischemic conditioning.

We tested monocyte-derived EVs (MEVs) targeting CD14, granulocyte-derived EVs (GEVs) targeting CD66b and EVs using the superficial epitope CD15 as a marker for both granulocytes and monocytes (LEVs). The course of MEVs was generally stable; however, we observed a significant steady increase of MEVs in the RIC group, which became statistically significant one month after STEMI (3677 (95%CI 266–7089), *p* for interaction = 0.0325, Figure 3A).

Regarding GEVs, RIC was associated with significantly higher levels of GEVs (0.42 (95%CI 0.04–0.81), *p* = 0.027, Figure 3B). Furthermore, we observed a significant increase in the proportion of PS^+^ GEVs 24 h after PCI (4.9% (95%CI 0.3–9.6), *p* = 0.039 Figure 3C), without any association with RIC (−1.6% (95%CI −5.0–1.8), *p* = 0.358). 

With respect to LEVs, there was no difference regarding the PS^−^ LEVs over time or between the RIC and the control group (all *p* > 0.05); however, there was a significant decline in the PS^+^ LEVs with time (Figure 3D); 24 h (−162 (95%CI −316–−8), *p* = 0.0431), four days (−141 (95%CI −309–27), *p* = 0.1055), one month (−191 (95%CI −355–−29), *p* = 0.0238). Furthermore, we observed a nadir regarding the ratio of PS^+^ and PS^−^ LEV at 24 h after PCI (−9.4 (95%CI −16.9–2.0), *p* = 0.0135).

### 3.4. EVs after RIC in Healthy Volunteers

To further determine potential influence of RIC on EV, we investigated changes in plasma EVs in five healthy volunteers before and immediately after RIC, aiming to avoid effects of comorbidities and acute STEMI on EVs. Table 2 summarized concentrations of circulating EV populations before and after the conduction of RIC. There was no effect of RIC on any investigated EV population.

## 4. Discussion

In this randomized, controlled clinical trial, we studied different populations of EVs in the acute and sub-acute phase of STEMI, as well as the potential influence of RIC on EVs for the first time. In addition, we investigated EVs from healthy volunteers undergoing the same RIC protocol. Our study sheds light on the role of cell-to-cell communication via EVs in this pathological state that is characterized by enhanced inflammatory and pro-thrombotic processes.

While the scientific community increasingly recognizes the importance of EVs and their role in cellular communication, a large amount of data is derived from bench experiments with known difficulties to translate their results into clinical practice. Therefore, studying EVs and their changes over time in patients is of utmost importance. Different studies have investigated different EV populations during acute myocardial infarction previously [26,27,28,29,30]. However, it can be seen as a main problem that challenges in the characterization of EVs, together with a lack of standardization between previous studies results in a reduced ability to compare studies. In this regard, our study aims at a standardized evaluation and followed previously published protocols [23,24]. In contrast to some previous investigations, we studied different EV sub-populations during the acute phase of STEMI (before and after reperfusion therapy), as well as up to one month after the acute event. As important parts of cardiomyocyte remodeling post STEMI are characterized by inflammatory processes and takes place during the first days and weeks after STEMI [31], the present study design also provides insights on the potential involvement of circulating EVs in this critical phase. Previous in vitro experiments could already demonstrate that the signature of EVs per se changes within the myocardium under ischemic conditions, of which larger EVs exert an inflammatory stimulus [32].

The present study reveals changes regarding CD66b and CD15 EV over time, which is in line with the simultaneous involvement of the releasing cells in cardiac remodeling [31]. In addition, the superficial expression of PS changes gradually in circulating EVs of STEMI patients. This observation is important insofar, as several studies used the superficial expression of PS to characterize EVs in general. However, the differences in the number of PS^+^ and PS^−^ EVs observed in our study strongly suggest different pathomechanistic properties of these EV populations, strengthening evidence that PS is not the ideal target for studying circulating EVs in general. With this respect, especially PS^+^ EVs have been shown to be exposed to several changes during acute myocardial infarction. We are able to show that the proportion of PS^+^ expression rises during the acute phase of STEMI, coinciding with the acute phase of reperfusion. As this parallels the time of active cellular damage during AMI, it remains unclear whether the increased number of PS^+^ EVs is only an expression of damage solely or has further pathomechanistic importance.

In contrast to previous studies [33], we found no significant difference regarding the course of EEVs during the acute phase after STEMI. Methodological differences compared to previous investigations, including the used antibodies, flow cytometer or the silica beads used to set the upper size limit, may account for this difference. However, EEVs significantly increase one month after the acute event. This is of importance, as previous studies were able to demonstrate a harmful effect mediated by EEVs. For instance, EEVs from patients with acute coronary syndrome induced endothelial aging and dysfunction [29]. Moreover, EEVs from patients with congenital heart disease were also associated with endothelial dysfunction [34]. Altogether, EEVs and their observed increase after STEMI could hypothetically be a therapeutic target in the future.

Our study primarily aimed to study the effects of RIC on circulating EVs in patients with STEMI. While several preclinical studies strongly support the cardioprotective concept of RIC, previous meta-analyses already questioned the effect of RIC in patients with AMI. Additionally, a recently published large randomized controlled trial did not show any effect on cardiac death or the rate of hospitalization for heart failure within one year in patients with STEMI [12,13]. As discussed previously, several reasons might explain this finding [35]. However, the present study was not designed to investigate differences regarding clinical events or infarct size in patients receiving RIC or a sham intervention.

Although the exact ways of actions of RIC have not been deciphered yet, the current concept of how RIC mediates cardioprotection involves a humoral and a neuronal pathway. As reviewed previously, intact neuronal pathways, particularly involving the vagal nerve, are important to initiate a cardioprotective signal [11]. Furthermore, cardioprotection by RIC may be transferred via a (unknown) humoral factor [11,36]. In this regard, EVs have been suggested to be involved in the mediation of cardioprotection as well. Previous studies reported an increased number of circulating EVs following the conduction of a similar RIC protocol, which was further associated with a cardioprotective effect against ischemia/reperfusion injury [15,16]. As summarized previously, several EV populations seem to have cardioprotective properties [37]. However, the present study is the first to investigate the effect of RIC on circulating EVs in STEMI. In line with the believe that RIC might not exert any, or if then insufficient biological effect in humans, we did not observe any influence of RIC on the investigated EV sub-populations in STEMI patients (with only two exceptions). This is true for EV measurements taken directly after patients did undergo RIC and PCI, as well as for the further course of EVs following STEMI. Since STEMI has impact on circulating EVs as reported by the present and previous studies [26,27,28,29,30], these effects have also not been profoundly affected by RIC. However, we did observe a significant increase of EEV in STEMI patients undergoing RIC. Since EEVs have been recognized in different processes in cardiovascular diseases and have been associated with impaired endothelial function, this finding should be addresses in future studies. On the other hand, the standard deviation is very high in this group, wherefore results need to be interpreted cautiously.

Additionally, we tested the same RIC protocol in healthy volunteers to better clarify any potential direct effects of RIC on circulating EVs. These participants were required to be free from any known diseases and were not allowed to take any drug on a regular basis. The study of RIC in healthy volunteers enables the possibility to investigate effect on EVs in more detail without confounding comorbidities or inflammatory and thrombotic processes how they occur during STEMI (and which are known to have influence on circulating EVs as discussed above). In line with our results derived from STEMI patients, we did not observe any significant change comparing circulating EVs before and after RIC.

### 4.1. Strengths and Limitations

Finally, our study has limitations. First, the sample size is rather small, wherefore definitive answers may not be drawn. However, our exploratory design will provide a basis for future investigations of EVs in STEMI patients. Consequently, the chance of deriving extreme results and of overinterpretation of formally statistically significant results has to be kept in mind upon interpretation of results. Second, even though efforts increased remarkably to standardize the studies of EVs and guidance is provided by international organizations [38], they are still highly heterogenous and comparability between studies is not guaranteed. For instance, the techniques of EV characterization will have distinctive influence on the results. In the present study we used a high-sensitive flow cytometer for all experiments, we used fluorochrome-triggering over scatter-triggering [39] at a low flow rate [23] to increase the number of EVs detected and we used silica beads over polystyrene beads due to their closer refractive index to biologic material to not overestimate the upper-size limit of EVs [40]. Nevertheless, the characterization of EVs using flow cytometry underlies several influences, including fluorochromes, cytometers, gating strategies, laser configuration and others, wherefore comparability will be challenging. Besides, flow cytometers per se will have limitations in the detection of the smallest EVs based on their technical setup. Finally, as the initial triggering signal will have a huge impact on the number of EVs detected, our study makes use of a dual marker strategy targeting the fluorochrome signal of Calcein AM and/or Lactadherin.

### 4.2. Conclusions

In summary, the present randomized controlled trial suggests that the conduction of a RIC protocol has no effect on the numbers of circulating EV in patients with STEMI. Differences between RIC and control were only observed for EEV one month after STEMI and granulocyte-derived EVs in general, although these results have to be interpreted with caution, considering a high standard deviation of measurements. In line, RIC had no effect on EVs when applied to healthy volunteers. Additionally, certain sub-populations of EVs expressing PS are susceptible to changes following STEMI that should deserve further attention in future clinical trials.

## Figures and Tables

**Table 1:** Baseline characteristics of included patients.

	Total (*n* = 32)	Control (*n* = 16)	RIC (*n* = 16)	*p*-Value
Age, mean (SD)	61.4 (13.7)	59.6 (13.1)	63.2 (14.5)	0.463
BMI, mean (SD)	28.4 (5.1)	27.3 (3.5)	29.6 (6.2)	0.194
Hypertension, *n* (%)	18 (56.2%)	6 (37.5%)	12 (75.0%)	0.033
Hyperlipoproteinemia, *n* (%)	9 (28.1%)	4 (25.0%)	13 (56.2%)	0.066
Diabetes mellitus, *n* (%)	1 (3.1%)	1 (6.2%)	0 (0.0%)	0.310
Smoking, *n* (%)				0.195
Previously	13 (40.6%)	4 (25.0%)	9 (56.2%)	
Continued	14 (43.8%)	9 (56.2%)	5 (31.2%)	
History of coronary artery disease, *n* (%)	2 (6.2%)	0 (0.0%)	2 (12.5%)	0.144
Ambulatory medication, *n* (%)				
Acetylsalicylic acid	7 (21.9%)	2 (12.5%)	5 (31.2%)	0.200
ACE/ARB	15 (48.4%)	4 (26.7%)	11 (68.8%)	0.019
Statin	2 (6.2%)	0 (0.0%)	2 (12.5%)	0.144
Beta blocker	7 (22.6%)	1 (6.7%)	6 (37.5%)	0.040
Culprit lesion, *n* (%)				0.686
Left main	1 (3.1%)	1 (6.2%)	0 (0.0%)	
Left anterior descending artery	17 (53.1%)	8 (50.0%)	9 (56.2%)	
Right coronary artery	11 (34.4%)	6 (37.5%)	5 (31.2%)	
Circumflex artery	3 (9.4%)	1 (6.2%)	2 (12.5%)	
P_2_Y_12_ Inhibitor, *n* (%)				0.388
Prasugrel	23 (71.9%)	13 (81.2%)	10 (62.5%)	
Ticagrelor	8 (25.0%)	3 (18.8%)	5 (31.2%)	
Clopidogrel	1 (3.1%)	0 (0.0%)	1 (6.2%)	
PCI with stenting, *n* (%)	31 (96.9%)	16 (100.0%)	15 (93.8%)	0.31
Initial TIMI flow 0/I, *n* (%)	23 (74.2%)	11 (68.8%)	12 (80.0%)	0.712
Final TIMI flow III, *n* (%)	32 (100%)	16 (100%)	16 (100%)	1.0
Peak cardiac troponin, median (IQR)	75 (34; 160)	46,6 (26; 142)	99 (59; 183)	0.214
Peak creatine kinase, median (IQR)	1865 (1022; 3400)	1465 (563; 2557)	2678 (1503; 3729)	0.152

ACE = angiotensin-converting enzyme inhibitor; ARB = angiotensinreceptor blocker; BMI = bodymass index; IQR = interquartal range; RIC = remote ischemic conditioning; SD = standard deviation; TIMI = thrombolysis in myocardial infarction.

**Table 2:** EV concentrations before and after RIC in healthy volunteers (*n* = 5).

	Before	After	*p*-Value
PS^+^ EVs	1227.5 (512.2)	1380.0 (625.6)	0.73
Platelet EVs	2805.0 (2105.7)	3305.0 (1757.3)	0.34
PS^+^ platelet EVs	1115.0 (864.5)	1190.0 (614.6)	0.87
Endothelial EVs	1075.0 (846.5)	895.0 (430.6)	0.42
PS^+^ endothelial EVs	105.0 (165.3)	35.0 (41.8)	0.29
Leukocytes EVs	1285.0 (957.3)	905.0 (575.4)	0.14
PS^+^ leukocyte EVs	230.0 (221.8)	260.0 (85.9)	0.76
Monocyte EVs	1150.0 (1262.1)	1060.0 (651.6)	0.88
PS^+^ Monocyte EVs	225.0 (211.4)	205.0 (157.5)	0.87
Granulocyte EVs	315.0 (255.3)	485.0 (351.6)	0.19
PS^+^ granulocyte EVs	0.0 (0.0)	5.0 (11.2)	0.37

## Supplementary Materials

Supplementary materials can be found at https://www.mdpi.com/2227-9059/8/7/218/s1.

## Abbreviations

EEV	Endothelial-derived extracellular vesicles
EV	Extracellular vesicle
GEV	Granulocyte-derived extracellular vesicles
LEV	Leucocyte-derived extracellular vesicles
IRI	Ischemia-reperfusion injury
MEV	Monocyte-derived extracellular vesicles
PCI	Percutaneous coronary intervention
PEV	Platelet-derived extracellular vesicles
PS	Phosphatidyl-serine
RIC	Remote ischemic conditioning
STEMI	ST-elevation myocardial infarction
