# Supplementary material for: Changes in Circulating Extracellular Vesicles in Patients with ST-Elevation Myocardial Infarction and Potential Effects of Remote Ischemic Conditioning—A Randomized Controlled Trial
Source: Biomedicines. 2020 Jul 16;8(7):218. doi: 10.3390/biomedicines8070218 (PMC7400268; doi:10.3390/biomedicines8070218)
Supplement: Supplementary file 1 [file biomedicines-08-00218-s001.pdf]

Online-only Supplementary Material

Changes in circulating extracellular vesicles in patients with ST-elevation myocardial infarction and potential effects of remote ischemic conditioning – a randomized controlled trial

Paul M Haller et al.

Supplementary Figure S1: Patient enrolment

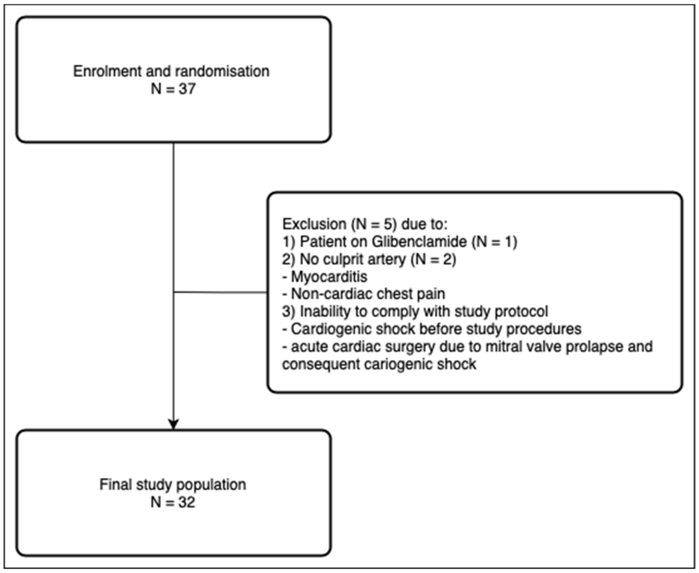

Blood sampling

Upon presentation to the emergency department with ST-elevation myocardial infarction (STEMI) patients were asked if they wanted to participate in the study. If patients gave written informed consent, blood was drawn using a 19 or 21 G needle and a vacutainer system with minimal stasis from a cubital vein. Further blood draws were scheduled for the first hour after percutaneous coronary intervention (PCI), as well as 24 hours, 4 days and 30 days thereafter. Each time, we used 4 ml EDTA plastic tubes and 3.5ml 3.2% citrate plastic tubes (Vacuette® tube, Greiner Bio-One, Kremsmünster, Austria) to collect blood for routine laboratory measurements and study proposes.

Blood handling

We only used citrate-anticoagulated blood for the analysis of extracellular vesicles (EV). In line with current recommendations (1), we performed two centrifugation steps: blood was centrifuged within 30 minutes after collection for the first time at room temperature with 2500xg for 20 minutes to generate platelet-poor plasma (PPP). PPP was collected and centrifuged again for 20 minutes at room temperature at 2500xg to generate platelet-free plasma (PFP). Aliquots of the PFP were immediately frozen at -80° Celsius until further use.

Labelling of extracellular vesicles

For EV analysis, PFP was thawed under controlled conditions in a water bath at 35° Celsius to avoid formation of ice crystals and reduce cryo-precipitation during this process. (2) We used the following anti-human monoclonal antibodies for all analyses: CD41-PC7 (clone P2), CD54-PE (clone 84H10), CD146-PE (clone TEA 1/34), CD11b-PE (clone Bear1), all from Beckman Coulter (Krefeld, Germany), CD14-PE/Cy7 (clone HCD14), CD15-PE/Cy7 (clone W6D3) or CD31-PE (clone WM50) all from Biolegend (CA, USA). We centrifuged all used antibodies at 20,000xg for 5 minutes before use to spin down undesired antibody conjugates.(3) For labelling, 10 µl of thawed PFP were diluted in 25 µl phosphate-buffered saline, which was filtered through a 0.2µm mesh before. Afterwards, antibodies in per-defined combination with a maximum of 2 different fluorochrome dyes per panel were added. To achieve maximal antibody binding we labelled EVs with antibodies for 90 minutes at room temperature in the dark.

Afterwards, we added 0.086µg bovine Lactadherin-Alexa 647 (Cell Systems, Troisdorf, Germany) targeting phosphatidyl serine (PS) and the intra-vesicular dye Calcein AM (Life Technologies, Carlsbad, CA). Samples were labelled with both for additional 30 minutes. Calcein AM per se is not fluorescence, but after transportation into intact EVs it undergoes an esterase-triggered conversion to calcein, which is a strong green florophore and may be detected by the flowcytometer. (4,5)

Flowcytometry-based detection and characterization of extracellular vesicles

All analyses were performed on a Cytoflex LX flow cytometer (Beckman Coulter, Krefeld, Germany) and were analyzed using the CytExpert software version 2.2. The flow cytometer was equipped with four lasers (375nm, 405nm, 488nm, 638nm, 561nm and 375nm, respectively). Daily maintenance using manufacture beads, washing steps and instrument calibration were performed in accordance with the manufacturer's recommended protocols. Furthermore, we performed additional washing steps prior to EV analyses to assure a clean system.

In line with current recommendations (1) and previously established protocols (4,6), we used fluorescence-triggering of signals over conventional side-scatter triggering for all analyses as it refined the detection of especially small EVs (7). The trigger signal was set on a positive fluorescence signal in the Lactadherin or Calcein AM channels. In addition, we used 1000nm Silica beads (Ksiker Biotech, Steinfurt, Germany) to set an upper size limit to define EVs. We choose Silica beads as their refractive index is closer to biological material wherefore estimation of EV size is better compared to Polysterene beads (8). Beads were detected in a violet-side scatter and forward scatter plot (Figure 3).

Gates for all antibodies were set corresponding isotype control antibodies (Figure 3). To assure the measurement of EVs, we also stained PBS without sample with antibodies and Calcein AM and introduced 2-minute washing steps with sterile water to avoid spill over. Finally, EVs of stained samples were destroyed using triton. EV characterization of these samples was not possible anymore (Figure 3).

Prior to characterization of all EVs, we performed dilution experiments to avoid swarm effects (9). In flowcytometry-based EV characterization, swarm effects may occur when several very small signals/particles/EVs pass the laser at the same time. The laser could not discriminate all the individual signals, wherefore one large signal would be detected. Finding the right dilution is, therefore, important prior to the analysis of the whole project. Finally, we kept the rate of flow on the flow cytometer at low speed and the number of detected events per second below 2000 as described previously (4).

A previously published study did not find differences regarding the enumeration of EVs comparing counting beads and the detected event rate on a Cytoflex S (4). Hence, we defined all EV populations as follow: $CD31^+/CD54^+/CD146^+/CD41^-$ for endothelial EVs (EEV), $CD41^+$ for platelet-derived EVs (PEV), $CD14^+$ for monocyte-derived EVs (MEV), $CD15^+$ for leukocyte-derived EVs (LEV) and $CD66b^+$ for granulocyte-derived EVs (GEVs), respectively. The gating strategy is also shown in Figure 1. EVs are given as number per µl of pure PFP.

Supplementary References:

1. Thery C, Witwer KW, Aikawa E et al. Minimal information for studies of extracellular vesicles 2018 (MISEV2018): a position statement of the International Society for Extracellular Vesicles and update of the MISEV2014 guidelines. J Extracell Vesicles 2018;7:1535750.
2. Coumans FAW, Brisson AR, Buzas EI et al. Methodological Guidelines to Study Extracellular Vesicles. Circ Res 2017;120:1632-1648.
3. Aass HC, Ovstebo R, Troseid AM, Kierulf P, Berg JP, Henriksson CE. Fluorescent particles in the antibody solution result in false TF- and CD14-positive microparticles in flow cytometric analysis. Cytometry A 2011;79:990-9.
4. Wisgrill L, Lamm C, Hartmann J et al. Peripheral blood microvesicles secretion is influenced by storage time, temperature, and anticoagulants. Cytometry A 2016;89:663-72.
5. Gray WD, Mitchell AJ, Searles CD. An accurate, precise method for general labeling of extracellular vesicles. MethodsX 2015;2:360-7.
6. Haller PM, Stojkovic S, Piackova E et al. The association of P2Y12 inhibitors with pro-coagulatory extracellular vesicles and microRNAs in stable coronary artery disease. Platelets 2019:1-8.
7. Arraud N, Gounou C, Turpin D, Brisson AR. Fluorescence triggering: A general strategy for enumerating and phenotyping extracellular vesicles by flow cytometry. Cytometry A 2016;89:184-95.
8. van der Pol E, Coumans F, Varga Z, Krumrey M, Nieuwland R. Innovation in detection of microparticles and exosomes. J Thromb Haemost 2013;11 Suppl 1:36-45.
9. van der Pol E, van Gemert MJ, Sturk A, Nieuwland R, van Leeuwen TG. Single vs. swarm detection of microparticles and exosomes by flow cytometry. J Thromb Haemost 2012;10:919-30.
